# The Choroid and Optical Coherence Tomography

**DOI:** 10.4274/tjo.10693

**Published:** 2016-01-05

**Authors:** Taha Sezer, Muhammet Altınışık, İbrahim Arif Koytak, Mehmet Hakan Özdemir

**Affiliations:** 1 Bezmialem Vakıf University Faculty of Medicine, Department of Ophthalmology, İstanbul, Turkey

**Keywords:** Choroid, optical coherence tomography, age-related macular degeneration, central serous retinopathy, degenerative myopia

## Abstract

The choroid is the most vascular tissue in the eye and it plays an important role in the pathophysiology of various common chorioretinal diseases such as central serous retinopathy, age-related macular degeneration and degenerative myopia. Quantitative assessment of the choroid has been quite challenging with traditional imaging modalities such as indocyanine green angiography and ultrasonography due to limited resolution and repeatability. With the advent of optical coherence tomography (OCT) technology, detailed visualization of the choroid in vivo is now possible. Measurements of choroidal thickness have also enabled new directions in research to study normal and pathological processes within the choroid. The aim of the present study is to review the current literature on choroidal imaging using OCT.

## INTRODUCTION

The choroid is a pigmented vascular tissue that was first histologically examined in the 17th century, and to date has been studied by various imaging methods. It extends from the ora serrata anteriorly to the optic nerve head posteriorly. According to histopathological examination, the choroid has a mean thickness of 0.15 mm anteriorly and 0.22 mm posteriorly; anatomically it forms the posterior portion of the uveal tract, which continues anteriorly with the ciliary body and the iris. From retina to sclera, the choroid comprises Bruch’s membrane, the choriocapillaris, the medium diameter choroidal vessels, and the large diameter choroidal vessels.^1^

A structurally and functionally healthy choroid is essential for retinal function. The central retinal artery nourishes the inner two-thirds of the choroid, whereas the choroidal vasculature nourishes the outer third. Abnormal choroidal circulation leads to retinal photoreceptor dysfunction and death.^2^ It has been shown that the choroid has a vital role in the pathophysiology of many diseases such as central serous retinopathy (CSR), age-related macular degeneration (AMD), pathological myopia and Vogt-Koyanagi-Harada (VKH) disease.^3,4,5,6^ Thus, the clear and accurate identification of choroidal changes will allow the proper assessment of many posterior segment diseases. However, unlike the retina, choroidal structures are not found in distinct, ordered layers and they lack contrasting reflective properties; therefore, for many years it was not possible to examine the choroid in as much detail as the retina.

## CHOROIDAL IMAGING METHODS

Until recently, the choroid was evaluated in vitro by histological analysis or in vivo by indocyanine green angiography (ICGA), laser Doppler flowmetry and ultrasonography.^7,8^ As histologic studies were done in conditions that did not preserve the tone of the vascular structures, the analysis was very limited in terms of understanding disease pathophysiology. The choroid of a lifeless eye is deflated, resulting in greatly underestimated thickness measurements. Furthermore, considering that many of the factors influencing vascular tone do not exert their effect in histological sections, it is clear that in vitro evaluation is insufficient.

ICGA allows the visualization of the choroidal vessels and circulation under the retinal pigment epithelium (RPE). It has been shown that ICGA is better than fundus fluorescein angiography (FFA) in showing the details of choroidal neovascularization (CNV) and detecting choroidal polyps. In addition, the longer wavelengths used in ICGA enable better visualization of underlying lesions in cases with blood, exudates and pigment epithelial detachment (PED).^9,10^

Laser Doppler flowmetry is a non-invasive diagnostic method that allows the evaluation of hemodynamic parameters of optic nerve head, iris and subfoveal choroidal circulation by determining the average speed and number of erythrocytes moving in a specific volume. Laser Doppler flowmetry has been used to show that choroidal circulation is decreased in diseases like diabetic retinopathy, AMD and retinitis pigmentosa.^11,12^

Ultrasonography has been an important diagnostic tool in situations where the posterior pole cannot be evaluated due to opacities in the anterior of the eye. In particular, it enabled the detection of thickening and tumors in the choroid and retina. However, because of its low image resolution, detecting small changes in the choroid is difficult, and it is not an ideal method for measuring a relatively thin tissue like the choroid.^13^

Although these three techniques were used for many years to detect choroidal vessel abnormalities and circulation changes, none of them provided in vivo cross-sectional images of the anatomy of the retinal pigment epithelium or choroidal layers, and none provided sufficient data regarding true choroidal thickness and morphology.

Optical coherence tomography (OCT) is a relatively new method that has enabled the acquisition cross-sectional images of the retina in a way similar to ultrasound, but at much higher resolution. Technically, OCT is a partial coherence interferometer. Coherent light refers to light of a single wavelength, like laser light. Partially coherent light includes light of different wavelengths within a narrow range. The partially coherent light used in OCT is of a specific wavelength provided by a laser source. The basic principle of OCT is similar to B-scan ultrasound, but by means of light instead of sound.^14^

The first OCT technology was time domain (TD) OCT, in which light from a light source passes through a semitransparent mirror called the beamsplitter. This mirror splits the beam in two and sends half to a reference mirror at a known and adjustable distance from the detector, and the other half into the eye. A tomographic cross-section of the tissue is created based on the temporal difference between the beams reflected from the reference mirror and the ocular structures with various reflective properties. With the most advanced and recently produced TD-OCT device, the Stratus OCT, it is possible to acquire an average of 400 A-scans per second and images at 10 µm resolution.^15^

The OCT technology currently used in routine practice is spectral domain (SD) OCT. Unlike TD-OCT, SD-OCT does not use a reference mirror; instead, light reflected from the various tissue layers is detected simultaneously by a high-speed spectrometer and processed with a Fourier transform. For this reason, SD-OCT is also known as Fourier domain OCT. The use of a spectrometer to detect light reflected from tissue allows SD-OCT instruments to acquire 20,000-52,000 A-scans per second and produce images with 5 µm resolution.^16^

The signal-to-noise ratio is used to express the quality of OCT images. The choroid cannot be visualized by TD-OCT due to its low signal-to-noise ratio. The hyperreflectivity of the RPE layer prevents light from reaching the choroid and therefore a return signal is not received from the deeper choroidal layers. The low resolution of TD-OCT also prevents the detailed visualization of the choroid. Although SD-OCT is better than TD-OCT for choroidal imaging, it is not possible to obtain images extending to the choroidoscleral junction. Imaging of the entire choroid by SD-OCT is also prevented by the posterior position of the choroid, the use of wavelengths too short to penetrate the choroid, and the aforementioned hyperreflectivity of the RPE. OCT devices use light at a wavelength of 800 nm, whereas a wavelength of 1060 is required for choroidal imaging. Increasing the wavelength results in lower image resolution and quality. Because of these limitations, even with SD-OCT instruments it was not possible to visualize the entire choroid at high resolution until the development of enhanced depth imaging (EDI) OCT technology.^17^

## ENHANCED DEPTH IMAGING-OPTICAL COHERENCE TOMOGRAPHY

In standard SD-OCT instruments, tissue depths are encoded by different frequencies of the interference spectrum. With increasing tissue depth, the reflected light echoes from the deep tissue layers travel farther to the detector located at the zero delay line. Because these echoes have lower signal strength and higher frequency, the spectrometer cannot differentiate them. Thus, in standard OCT, the proximity of the retinal structures to the zero delay line results in high-sensitivity images of the retina and vitreoretinal junction and decreasing sensitivity toward the choroid. Two ways to overcome this are increasing the sensitivity of the spectrometer for high frequency detection or increasing the pixel number on the camera at the zero delay line. However, the image resolution of standard OCT cannot be increased and desired image quality cannot be achieved with these techniques. Therefore, specific protocols are used for choroid imaging. One of these protocols involves taking multiple images from the same area of the retina, increasing the signal-to-noise ratio, and combining them to produce an averaged image. This technique has been integrated as software into many SD-OCT instruments. Depending on the type of device, 8 to 100 images can be obtained and merged. Eye movement tracking systems have been developed to improve the image quality and signal strength while taking simultaneous images from the same location. However, even with this protocol it has not been possible to acquire choroid images of the desired quality.^14^

**Figure 1:**
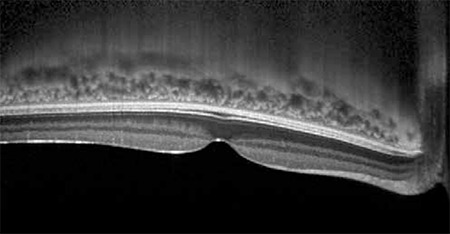
The choroid appears as an inverted image on optical coherence tomography imaging without enhanced depth imaging-optical coherence tomography mode. This image was captured using the Spectralis spectral domain-optical coherence tomography device (Heidelberg Engineering, Heidelberg, Germany)

Another protocol emerged as the result of a technical characteristic of SD-OCT devices. SD-OCT cannot distinguish between positive and negative echoes; therefore, if a retinal structure is brought closer to the zero delay line, after a certain point positive echoes become negative echoes. When the detector produces an image from these echoes, an inverted mirror image appears reflected across the zero delay line. If the device is advanced toward the eye, an inverted SD-OCT image of the choroid can be captured on the screen (Figure 1). Because the choroid is closer to the zero delay line, its echoes have high signal strength and low frequency, increasing the resolution of the choroidal image. This technique was first described by Spaide et al.18 as EDI, and has been integrated as software into SD-OCT devices.

## ENHANCED DEPTH IMAGING-OPTICAL COHERENCE TOMOGRAPHY CAPTURE TECHNIQUE

The EDI-OCT imaging technique does not require any changes to the hardware of SD-OCT devices. As previously described, the technique consists of bringing the choroid closer to the zero delay line to focus the device more posteriorly in the optical system and processing the acquired images via software. Therefore, choosing EDI-OCT mode in the user interface of the original or updated software for choroidal imaging is sufficient. The patient is then asked to look at the reference light and images are acquired as in standard macular imaging. Dilating the pupil before imaging is not necessary.

The most useful application of choroidal imaging with EDI-OCT reflected in clinical practice is the detection of choroidal thickness. Although this measurement is currently performed manually on all devices, the intervisit, interobserver and intersystem agreement is very good, and highly reliable measurements can be obtained. The most common measurement used in daily practice is subfoveal choroidal thickness, which is detected by using the software’s caliper tool to draw a line connecting the outer border of the retinal pigment epithelium and the inner border of the sclera. Care should be taken that this line is perpendicular to the line tangential to the foveal contour. The same tool can also be used to take thickness measurements at different points in the choroid.

## ENHANCED DEPTH IMAGING-OPTICAL COHERENCE TOMOGRAPHY AND THE NORMAL CHOROID

**Figure 2:**
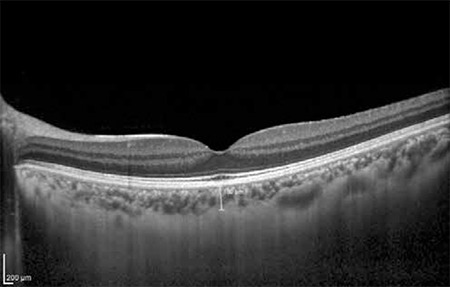
Choroidal thickness measurement at the fovea in a healthy eye. Choroidal thickness is measured manually as the horizontal distance between the outer edge of the hyperreflective retinal pigment epithelium and the inner edge of the choroidoscleral junction

**Figure 3:**
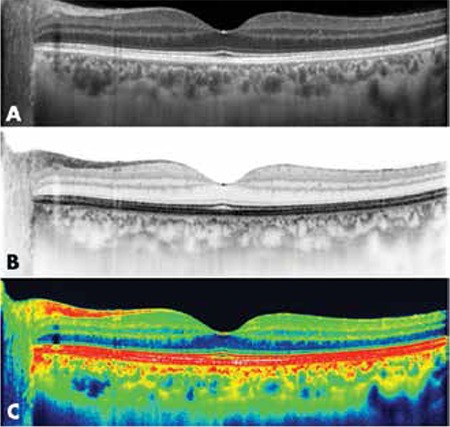
Black-over-white (A) optical coherence tomography images are preferrable to white-over-black (B) or colored (C) optical coherence tomography images for visualization of the choroid

Normal choroidal thickness was first described by Margolis and Spaide^19^ using the Spectralis (Heidelberg Engineering, Heidelberg, Germany) and Manjunath et al.^20^ using the Cirrus HD-OCT (Carl Zeiss Meditec, Dublin, CA, USA). Choroidal thickness was measured manually as the distance perpendicularly between the hyperreflective outer edge of the RPE to the choroidoscleral junction (Figure 2). It may not be possible to detect the choroidoscleral junction in some eyes. For this reason it is recommended to obtain as clear images as possible, and black-over-white OCT images are preferable to white-over-black or colored OCT images (Figure 3). As a result of their examinations, they found that the choroid was thickest under the fovea and thinner at the nasal retina compared to the temporal. Choroid thickness decreases with distance from the fovea. The reason for the maximum subfoveal choroidal thickness is to meet the high oxygen demand of the retinal cells at the fovea. Subfoveal choroidal thickness was measured as 287±76 µm (54 eyes of 30 patients) with the Spectralis and 272±81 µm (34 eyes of 34 patients) with the Cirrus. Both groups showed a negative correlation between choroidal thickness and age.^20^ In other words, the choroid thins with increasing age. However, we predict that larger studies of choroidal thickness and the effect of aging on choroidal thickness in normal eyes will yield more quantitative data about this thinning. Furthermore, choroidal thickness of the same individual can vary between measurements taken at different times.^21^

Reproducibility and reliability are basic prerequisites for the utilization of any technique. In EDI-OCT, choroidal thickness measurements are done manually, not automatically. Ikuno et al.^22^ studied the reliability and reproducibility of manual normal choroidal thickness measurements on EDI-OCT. In their study, the choroidal thickness of 10 volunteers was measured by 6 different people twice with an interval of 4 months. Interobserver correlation was found to be 0.970 (95% CI, 0.948-0.985) and intervisit correlation was 0.893 (95% CI, 0.864-0.916). In a study comparing the Cirrus, Spectralis and Optovue (Optovue, Inc, Fremont, CA, USA), choroid thickness at five different locations was measured with each of the three devices, and the measurements were strongly correlated (p<0.0001).^23^

To accurately evaluate choroidal pathologies, normal choroidal morphology on EDI-OCT must be determined in addition to normal choroidal thickness. In a study including 42 subjects, all were shown to have a bowl-shaped choroidoscleral junction, 98.8% of the patients’ choroidal vessels were distributed along the nasal-temporal axis, and large choroidal vessels comprised 80% of the subfoveal choroidal thickness.^24^

**Figure 4:**
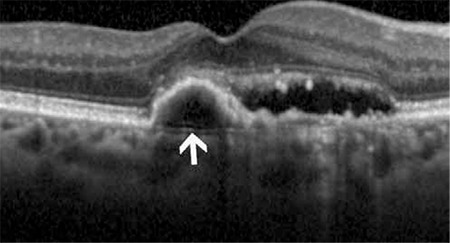
Bruch’s membrane (white arrow) in an age-related macular degeneration patient with pigment epithelial detachment

**Figure 5:**
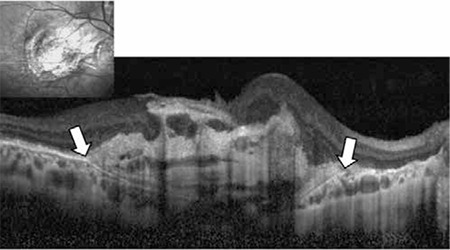
Thickening of Bruch’s membrane and breaks in its hyperreflective line (white arrows) in a patient with angioid streaks

**Figure 6:**
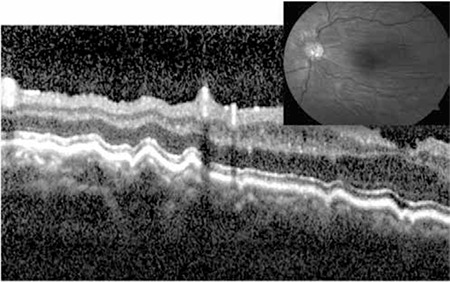
Enhanced depth imaging-optical coherence tomography reveals folding of Bruch’s membrane in hypotony maculopathy

With current OCT technology, it is not possible to see Bruch’s membrane, the first superficial layer of the choroid immediately under the RPE, in normal eyes. The echoes from this tissue cannot be differentiated from the strong signals coming from the RPE. However, in eyes where the RPE has separated from Bruch’s membrane (as in PED due to AMD or CSR), Bruch’s membrane appears as a thin line of mild hyperreflectivity (Figure 4). Angioid streaks have a known basis in collagen tissue disease, and the histological changes they cause in Bruch’s membrane have been known for many years. EDI-OCT of cases with angioid streaks reveals thickening of Bruch’s membrane and interruptions in its hyper-reflective line (Figure 5). Folding of Bruch’s membrane has been clearly shown on EDI-OCT in hypotony maculopathy (Figure 6).^25^

**Figure 7:**
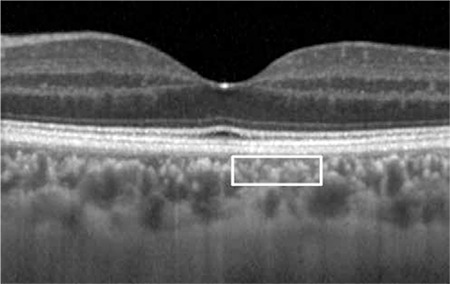
Beneath the hyperreflective retinal pigment epithelium, medium diameter choroidal vascular structures typically appear as two to four rowls of small, hyperreflective spots (white box). This layer is sometimes referred to as Sattler’s layer

**Figure 8:**
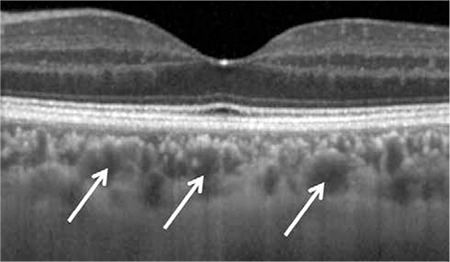
Choroidal arteries and veins are indicated with white arrows. This layer comprises the largest choroidal vessels and is also called Haller’s layer

The choroidal network of capillaries (choriocapillaris) found beneath Bruch’s membrane cannot be visualized using current OCT technology. However, the next layer contains mid-sized vessels (choroidal arterioles and venules), which appear as two to four rows of small hyperreflective spots immediately beneath the hyperreflective line of the RPE layer (Figure 7). This layer is referred to as Sattler’s layer in some sources, which usually portray it as the first 20-30 microns of choroid under the RPE.^25^ The largest vessels, the choroidal arteries and veins, can be distinguished as round or oval shapes deeper in the choroid (Figure 8). This layer is also called Haller’s layer.

## ENHANCED DEPTH IMAGING-OPTICAL COHERENCE TOMOGRAPHY AND CHORIORETINAL DISEASES

### Central Serous Chorioretinopathy

Central Serous Chorioretinopathy (CSC) is a condition characterized by exudative detachment of the neurosensory retina, and is usually seen in young men. In the acute stage, it appears on OCT as subretinal fluid accumulation and PED. Studies of CSC using ICGA have revealed hyperpermeability of the choroidal vessels that is much greater than expected based on RPE leakage seen on FFA. It is believed that this increased permeability of the choroidal vasculature and RPE barrier dysfunction play an important role in the pathophysiology of the disease.^26^

**Figure 9:**
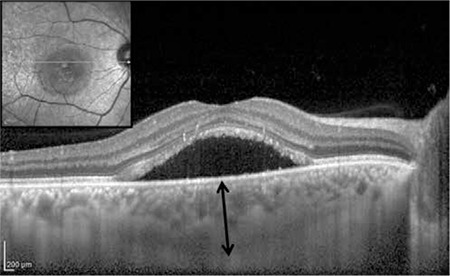
Thickened choroid in an eye with central serous retinopathy. Subfoveal choroidal thickness (black arrow) is 542 microns

As the primary pathology in CSC is in the choroid, EDI-OCT is especially important in the diagnosis of the disease (Figure 9).^27^ It has also been shown that patients with unilateral CSC have increased choroidal thickness in the unaffected eye,^28^ which suggests that there may be bilateral elevated hydrostatic pressure in the choroidal vasculature in CSC. In a comparison of photodynamic therapy and laser photocoagulation in patients with chronic CSC, it was shown with EDI-OCT that photodynamic therapy resulted in a decrease in choroidal thickness, whereas this effect was not observed after laser photocoagulation.^29^ Therefore, EDI-OCT can be used in the differential diagnosis of CSC from serous retinal detachment and in the follow-up of CSC patients. Furthermore, we believe EDI-OCT data are valuable in the identification of patients that may show susceptibility to CSC, which is important in order to warn these patients of factors that may trigger CSC attacks.

## AGE-RELATED MACULAR DEGENERATION

AMD is one of the leading causes of blindness in individuals over 60 years old, especially in developed countries.^30^ OCT is currently the imaging method most frequently utilized in the diagnosis, treatment planning and follow-up of AMD patients. OCT enables detailed detection of changes in the retina and RPE layer of AMD patients and even allows the identification of disease subtypes. As with CSC, it is currently widely accepted that the choroid is the primary tissue responsible for AMD. Therefore, evaluation of the choroid by EDI-OCT is of major importance in AMD patients; there are hundreds of studies in the literature on this topic.

**Figure 10:**
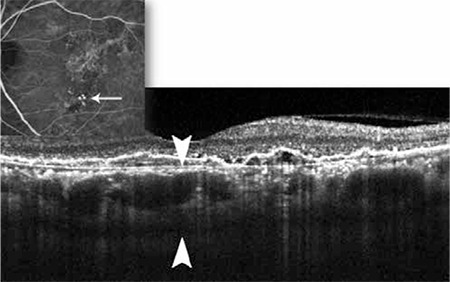
Thickened choroid in polypoidal choroidal vasculopathy

In practical terms, the main application of EDI-OCT in AMD is the recognition of patients with polypoidal choroidal vasculopathy (PCV). Several studies using EDI-OCT have shown greater subfoveal choroidal thickness in PCV compared to other AMD subgroups (Figure 10).^31^ This choroidal thickening occurs due to dilation of the mid-sized and large choroidal vessels and an increase in the choroidal vascular permeability seen on ICGA. This OCT finding is crucial because PCV, the frequency of which has not been determined in Turkey, is known to be resistant to anti-VEGF injection, the accepted standard treatment for AMD. PCV cannot be detected by FFA; the polypoidal structures of PCV can be detected with a difficult and expensive procedure like ICGA. Therefore, PCV must be considered upon observation of a thick choroid in AMD patients. Other important OCT findings that support a PCV diagnosis are the presence of RPE elevations similar but sharper than those found in PED with neighboring RPE irregularities.^25^

Another advantage of EDI-OCT in AMD is the opportunity to examine the interior of PED. In a study of 22 eyes, Spaide^32^ showed regression of a hyper-reflective structure within the PED after anti-VEGF therapy, suggesting that CNV is present and leads to the formation of PED. EDI-OCT was used in another study to detect choroidal thickness changes in AMD patients after anti-VEGF therapy.^33^ These studies may be valuable for guiding anti-VEGF treatment protocols, especially when discussing a side effect like the development of geographic atrophy.

EDI-OCT also enabled the identification of age-related choroidal atrophy, the basic pathology of which is advanced atrophic choroid. Age-related choroidal atrophy is characterized by an extremely thin choroid (without high myopia), senile sclerotic glaucoma, macular pigmentary changes, and severe impairment of near vision compared to far vision. The condition is generally seen in patients between 70 and 80 years old, and EDI-OCT is the principal diagnostic method.^4^

## DEGENERATIVE MYOPIA

Myopia is a refractive error resulting from a discrepancy between the eye’s optic power and its length. Degenerative myopia is a pathological condition with progressive lengthening of the eye, thinning of the sclera and choroid, and accompanying degenerative changes in the retina and RPE. Macular CNV and retinal detachment are frequently seen in these eyes and may lead to severe vision loss.^34^

**Figure 11:**
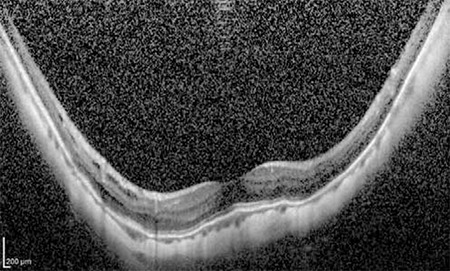
Vertical optical coherence tomography sections reveal extremely thin choroid in an eye with refractive error of -14.00 diopters

The most important OCT finding in degenerative myopic eyes is an extremely thin choroid (Figure 11). Fujiwara et al.^35^ demonstrated with EDI-OCT that choroidal thickness was significantly decreased in patients with high myopia. They also found that choroidal thickness was negatively correlated with age, refractive error, and presence of CNV. Myopic CNV causes less leakage than CNV in AMD and can be treated with less invasive therapies because choroidal vasculature supplying the CNV is not atrophic. Thus, much less subretinal accumulation is seen in myopic CNV compared to cases of AMD, which reduces the ability of OCT to show activity in myopic CNV.^25^

## GLAUCOMA

EDI-OCT is an important diagnostic method for understanding the vascular pathogenesis of glaucoma. Both macular and peripapillary choroidal thinning are used to investigate this pathogenesis. In studies of normal peripapillary macular thickness measurements, the inferior quadrant was significantly thinner than the superior quadrant, and the nasal quadrant was significantly thinner than the temporal quadrant.^36^

Maul et al.^37^ used EDI-OCT to evaluate whether choroidal thickness was associated with parameters such as age, axial length and nerve fiber layer, and found no significant difference between the peripapillary and macular choroidal thicknesses of patients with suspected glaucoma and patients with a glaucoma diagnosis. Many other studies have also shown that glaucoma does not affect choroidal thickness.^38,39,40^

The lack of significant changes in the macular choroidal thickness of glaucoma patients with severe visual field and retinal nerve fiber layer loss supports the view that there is no association between glaucoma and the macular choroid. Even in unilateral glaucoma cases there is no difference in macular choroidal thickness between the two eyes, which allows the elimination of systemic factors.

## DIABETIC RETINOPATHY

Our clinical experience suggests that choroidal vasculopathy is an important factor in the pathogenesis of diabetic retinopathy. Some histopathologic studies of diabetic eyes have shown various choroid pathologies like choroidal anomalies, choriocapillaris occlusion, CNV and choroidal aneurysm.^41^

Particularly in type 2 diabetes, choroidal thinning has been found in all eyes independent of the severity of retinopathy.^42^ This supports the reduced speed of the choroidal circulation previously demonstrated by laser Doppler flowmetry and ICGA.^43^ Because the choroid is the principal vascular structure nourishing the outer retinal layers and the RPE, choroidal thinning can result in hypoxia of the retinal tissues.

## VOGT-KOYANAGI-HARADA SYNDROME

OCT allows a noninvasive evaluation of uveitis patients, in whom investigation of the posterior segment is particularly challenging; therefore, SD-OCT is gaining popularity in the examination of uveitis patients.^44^

VKH is bilateral granulomatous panuveitis associated with autoimmunity against melanocytes. It is characterized by bilateral anterior and posterior uveitis with exudative retinal detachment.^45^ Increased choroidal thickness is seen in VKH, likely due to the inflammatory process and exudation.^46^ A significant reduction in the choroidal thickness of VKH eyes has been shown within two weeks of steroid treatment.^47^ Choroidal thickness evaluation is important for the assessment of treatment efficacy and the follow-up of recurrences.

## CONCLUSION

The EDI-OCT technique used with SD-OCT devices enables in vivo cross-sectional visualization of the choroid that is simple, reproducible, high-resolution and noninvasive, and has provided a better understanding of the choroidal changes that occur in many pathologies. However, this technique also has some limitations. One of the major drawbacks of the technique is that software allowing automated measurement of the choroid has not yet been developed. Manual measurement takes time and can result in inaccuracies. Eye tracking systems are necessary on OCT devices to ensure that the simultaneous images are taken at the same position. Another shortcoming of EDI-OCT is that in certain eyes, especially those with media opacities, relatively clear images of the retina can be obtained but the choroidoscleral junction cannot be clearly visualized.

The continuing search for new choroidal imaging technology has led to both software and hardware innovations like swept source OCT, Doppler OCT, long wavelength SD-OCT and en face OCT, and will remain a topic of interest for many years to come.
